# Modern surgical management of tongue carcinoma - A clinical retrospective research over a 12 years period

**DOI:** 10.1186/1758-3284-3-43

**Published:** 2011-09-29

**Authors:** Majeed Rana, Asifa Iqbal, Riaz Warraich, Martin Ruecker, André M Eckardt, Nils-Claudius Gellrich

**Affiliations:** 1 Department of Oral and Maxillofacial Surgery, Hannover Medical School, Hannover, Germany; 2 Department of Oral and Maxillofacial Surgery, King Edward Medical University, Lahore, Pakistan

**Keywords:** tongue cancer, squamous cell carcinoma, resection, survival, prognostic factors

## Abstract

**Objectives:**

In this retrospective study, we present a clinical review of our experience with tongue cancer in order to obtain valid criteria for therapeutic decision-making.

**Materials and methods:**

Between August 1999 and June 2011, a total of 398 patients with squamous cell carcinoma of the tongue were treated at the Department of Oral and Maxillofacial Surgery, King Edward Medical University Lahore Pakistan. Data concerning patient characteristics, clinical and pathologic tumour characteristics and treatment strategies and their results were obtained from a retrospective review of medical records. The average follow-up was 4.6 years. Statistical analysis for survival was calculated by the method of Kaplan and Meier.

**Results:**

There were 398 total patients. The mean age at diagnosis was 49.5 years,. 224 (56.3%) were male and 174 (43.7%) female (male/female ratio = 1.3:1).332/398 patients received surgical treatment, whereas 66 patients were excluded from surgical treatment and received primary radio (chemo) therapy after biopsy. Tongue carcinoma patients treated by non surgical treatment modalities had 5 years survival rate of 45.5% and patients with surgical intervention had survival rate of 96.1%.

**Conclusions:**

We recommend categorical bilateral neck dissection in order to reliably remove occult lymph node metastases. Adjuvant treatment modalities should be applied more frequently in controlled clinical trials and should generally be implemented in cases with unclear margins and lymphatic spread.

**Clinical relevance:**

This study provides modern treatment strategies for the tongue carcinoma.

## Introduction

Oral cancer located in the mouth, tongue or oropharynx is a significant health problem throughout the world. It's the eight most common cancer worldwide with 300.000 new cases reported annually [1]. Many countries feature incidence rates in oral cancer that vary in men from 1 to 10 cases per 100 000 population [2]. Developing countries suffer from higher incidence rates in oral cancer than developed countries [3]. Worryingly, the incidence of the disease is reportedly rising in most countries such as central and Eastern Europe and the USA [2,3]. The overall five-year-survival rate for patients with oral cancer stagnated for the last 20 years [4]. The survival rate is only 54% in industrial countries, one of the lowest rates of all major cancers. Five-year survival rates in developing countries reached the rate of 30% hardly [5]. The middle east is geographically located in the high incidence and mortality of oral cancers. Oral cancer is the second most common malignancy in both genders in Pakistan [1] and there is an epidemic alert of Oral cancers in Pakistan in the year 2030 by WHO [2].

First report of the tongue in medical literature was in 1635 [3]. But Only a limited number of studies have examined larger series of tongue cancer. Spiro and Strong evaluated 314 patients (1957-1963) with tongue cancer and found an overall 5-year survival rate of only 42% [3].

The incidence of tongue carcinoma in male is 6.5 per 100 000 per annum and in some parts of Europe and South Asia is up to 8.0 per 100 000 per annum. The tongue remains the most common intraoral site for oral cancer worldwide [4].

In contrast to other sites of oral cancer the incidence of the tongue carcinoma increasing in especially younger age group [5-7]. This is linked with Human papilloma etiology of tongue cancers [8]. This increase in the incidence needs more expertise and sharing of the experiences of the tongue carcinoma.

The optimum structural and functional integrity of this muscular organ of the Human body is vital for the life of the suffering patients. The speech, swallowing and breathing is associated with integrity of the reconstructed tongue muscles after surgical resection [9]. The anatomical and physiological milking muscle action predispose to an early invasion and metastasis of tongue carcinoma [10]. This results in extensive resection of not only the tongue tissue but also floor of mouth, oropharynx, tonsillar area along with cervical lymph nodes dissection even in clinical N0 status for the complete palliation of the occult metastasis [11].

The various treatment options for the tongue carcinoma include Surgery, radiotherapy, chemotherapy and combined Modalities [12]. Due to the mutilating affects of the surgical management of tongue carcinoma on the quality of life, organ preservation techniques and treatment protocols have been discussed. The choice of the treatment depends upon tumor factors such as site, size (T stage), location and multiplicity, proximity to bone, pathological features, histology grade and depth of invasion. The patient factors include status of cervical lymph nodes, previous treatments medical condition of the patient. The various flaps for mobile tongue include local (mucosal, Buccinator flaps), local neck flap(infrahyoid),free flaps (forearm free flap, antero-lateral thigh flap); For the base of tongue local neck flap (infrahyoid), free flaps (Latissimus dorsi free flap, Antero-lateral free flap, Rectus-abdominis free flap). The micro-vascular flap revolutionised the reconstruction of tongue and it was used first time in 1963 [13] in general surgery and in head and neck reconstruction in 1975 [14].

The resection defect classification guides clinicians for the decision of the reconstructive flap design. According to Urken et al tongue defects are difficult to classify; the volume and function of residual tissue does the quantification of the defect. He classified tongue resection defects as soft tissue defects of mobile tongue T^M^, base of tongue T^B ^and total glossectomy T^G ^defects along with neural defects. Further classification of T^M ^is done by longitudinal division in quarters and finally grouping of defects with reconstructive guidelines is described [15].

The purpose of the present study was to give a precise description of our experience with surgical based therapy of tongue cancer during 12 years in a country with limited Human expertise and finances. Furthermore, prognostic factors for survival were analyzed in order to obtain valid criteria for therapeutic decision-making in clinical routine.

## Materials and methods

Between August 1999 and June 2011, a total of 398 patients with squamous cell carcinoma of the tongue were treated at the Department of Oral and Maxillofacial Surgery, King Edward Medical University Lahore Pakistan. Data concerning patient characteristics, clinical and pathologic tumour characteristics and treatment strategies and their results were obtained from a retrospective review of medical records. The average follow-up was 4.6 years. Statistical analysis for survival was calculated by the method of Kaplan and Meier. The relationship between the clinic-pathologic variables and survival was assessed in univariate analysis using the log rank test. A value of p ≤ 0.05 was considered of to be statistically significant.

## Results

There were 398 patients according to the including criteria. The mean age at diagnosis was 49.5 years, ranging between 13 and 80 ± 10.6 years. There were 224 men (56.3%) and 174 (43.7%) women (male/female ratio = 1.3:1). The lesion size was T1 19/398 (4.8%), T2 60/398 (15.1%), T3 182/398 (45.7%) and T4 137/398 (34.41%) (Table 1). The primary site was lateral border of the mobile tongue 262/398 (65.8%), dorsum of tongue 36/398 (9.04%), base of the tongue 72/398 (181%) and all tongue involvement 28/398 (7.03%). Midline extension was seen in 128/398 (32.2%) of cases. Histopathologically 287/398 (72.1%) were well differentiated, 76/398 (19.1%) moderately differentiated, 12/398 (3%) were poorly differentiated, 12/398 (3%) were verrucous variants of squamous cell carcinoma and 11/398 (2.8%) were minor salivary gland malignancies (Table 2).

**Table 1:** Tumour size (T-Status) of patients and surgical treatment and survival rates of patients with surgical and non surgical management

			Tumour size				Total	Percentage
			T1	T2	T3	T4		
**No surgical management only radio chemotherapy**	**Years of survival**	1 year	0	7	3	6	16	24.2
		2 year	1	2	3	7	13	19.7
		3 year	0	0	1	3	4	6.1
		4 year	0	0	7	8	15	22.7
		5 year	0	0	7	11	18	27.3
	**Total**	**count**	1	9	21	35	66	100
		**Percent**	1.5%	13.6%	31.8%	53.0%	100%	
**Surgical management**	**Years of survival**	1 year	0	2	16	13	31	9.3
		2 year	2	5	22	17	46	13.9
		3 year	4	12	22	11	49	14.8
		4 year	3	14	38	24	79	23.8
		5 year	9	18	63	37	127	38.3
	**Total**	**count**	18	51	161	102	332	100
		**Percent**	5.4%	15.4%	48.5%	30.7%	100%	

**Table 2:** Histopathological variants with survival rates of Tongue carcinoma patients in surgical and non surgical treatment options

Treatment	Survival in years	Histopathology					Total
		Well differentiated SCC	Moderately differentiated d SCC	Poorly differentiated SCC	Verrucous SCC	Basisq uamou s SCC	
No surgical management	1	9	4	0	1	2	16 (24.2%)
	2	7	4	1	0	1	13 (19.7%)
	3	2	0	1	1	0	4 (6.1%)
	4	12	2	0	1	0	15 (22.7%)
	5	11	6	0	1	0	18(27.3%)
							
	Total	41 62.1%	16 24.2%	2 3.0%	4 6.1%	3 4.5%	66 100.0%
Surgery done	1	25	5	0	0	1	31 (9.3%)
	2	34	10	0	0	2	46 (13.9%)
	3	34	11	1	3	0	49 (14.8%)
	4	60	13	2	1	3	79(23.8%)
	5	93	21	7	4	2	127(38.3%)
							
	Total	246 74.1%	60 18.1%	10 3.0%	8 2.4%	8 2.4%	332 100.0%

332/398 patients received surgical treatment, whereas 66 patients were excluded from surgical treatment and received primary radio (chemo) therapy after biopsy. These patients refused surgery, were in inappropriate condition for general anaesthesia or suffered from inoperable tumour disease. As a consequence, the proportion of advanced tumour stages was higher in this group (Table 1). 317/398 (79.6%) had no previous history of premalignant oral lesion/condition where as 69/398 (17.3%) had the history of Oral premalignant lesion.12/398 (3%) had recurrence of the disease (Table 3).

**Table 3:** Previous history of Tongue carcinoma patients with their survival rate in surgical and non surgical treatment options

			Previous history			
			No history	H/O PML	H/O PMC	Recurrence
**No surgical management only radio chemotherapy**	**Years of survival**	1 year	9	2	2	3
		2 year	5	2	2	4
		3 year	2	0	1	1
		4 year	13	0	0	2
		5 year	9	4	2	3
	**Total**	**Count**	38	8	7	13
		**Percent**	57.6%	12.1%	10.6%	19.7%
**Surgical management**	**Years of survival**	1 year	21	9	0	1
		2 year	44	1	1	46
		3 year	38	10	0	1
		4 year	62	8	1	8
		5 year	102	22	1	2
	**Tota**	**Count**	267	50	2	13
		**Percent**	80.4%	15.1%	0.6%	3.9%

In patients with surgical therapy, the neck was staged pN0, pN1, pN2 and pN3 in 49.5%, 18.4%, 14.9% and 0.3% of cases. Supra-omohyoid neck dissection was done in 212/398 (53.3%) of the patients where as radical neck dissection in 88/398 (22.1%), bilateral neck dissection in 17/398(4.3%) patients; 15/398 (3.8%) had no neck dissection (Table 4). The primary closure was done in 38/398 (9.5%), local Myomucosal in 28/398(7%), Delto-pectoral in 138/398 (34.7%), Radial forearm free flap 100/398 (25.1%), Anterior thigh flap 16/398 (4.%), Rectus abdominis 12/398 (3.0%) (Table 5).

**Table 4:** Neck dissection and survival rate in tongue carcinoma patient

*Survival Years*	*Non Surgical *	*Neck dissection *				
		Supra omohyoid	Radical	Bilateral	Local excision	Total
1	16	19	9	3	0	31
2	13	28	14	2	2	46
3	4	30	13	2	4	49
4	15	48	25	3	3	79
5	18	87	27	7	6	127
**Total**	**66**	212	88	17	15	332
**Survival rate (%)**		63.9%	26.5%	5.1%	4.5%	100.0%
	**45.5**		**96.1**			

**Table 5:** Surgical options and their survival in Tongue carcinoma patients

Survival Years	Non Surgical		Surgical Management				
		
		Primary closure	Myo-mucosal flap	Delto-Pectoral	Radial FFF	Ant. Thigh	Rectus Abdominis
1	16	2	0	14	12	2	1

2	13	4	5	18	15	1	3

3	4	10	3	22	9	3	2

4	15	6	11	34	24	4	0

5	18	16	9	50	40	6	6

**Total**	**66**	**38**	**28**	**138**	**100**	**16**	**12**

**Survival rate (%)**	**45.5**	**94.7**	**100**	**94.9**	**97**	**93.8**	**100**
		
		**96.1**					

The par-operative frozen section technique for the margin free of tumour cells was done in 68/398(17.1%) patients; whereas histopathological reported tumour cell positive margins were observed in 56/398 (14.1%) and tumour cells negative margins were seen in 208/398(52.3%). Neo adjuvant radiochemotherapy was done in 10/398 (2.5%), adjuvant in 198/398 (49.7%) whereas 124/398 (31.2%) had no radiochemotherapy.66/398 (16.6%) were managed by radiochemotherapy without surgical intervention.

Overall 5 year survival rate was 349/398 (87.7%). The survival rate was calculated with Kaplan Meier Log rank with tumour size, treatment modality, previous history of the patient, histopathological variant, neck dissection options and radiochemotherapy modality.

**Figure 1:**
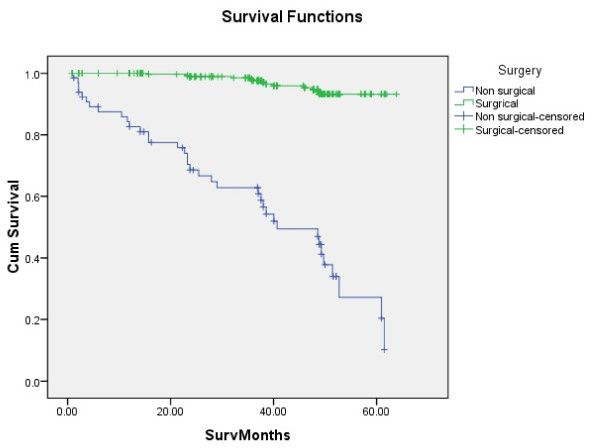
**Survival of patients with surgical treatment and non surgical management (log rank p < 0.001)**.

**Figure 2:**
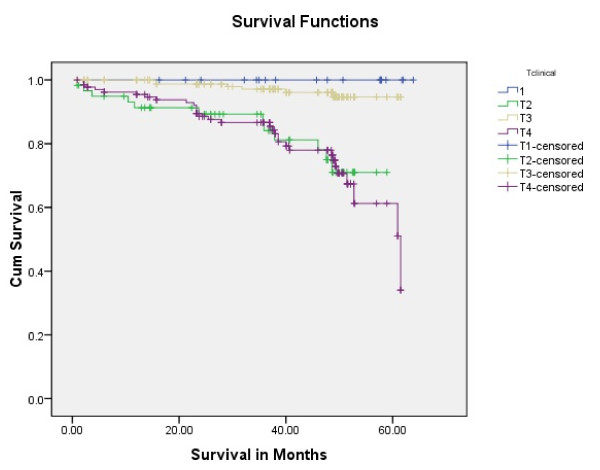
**Survival of patients with different tumour size (log rank p < 0.001)**.

**Figure 3:**
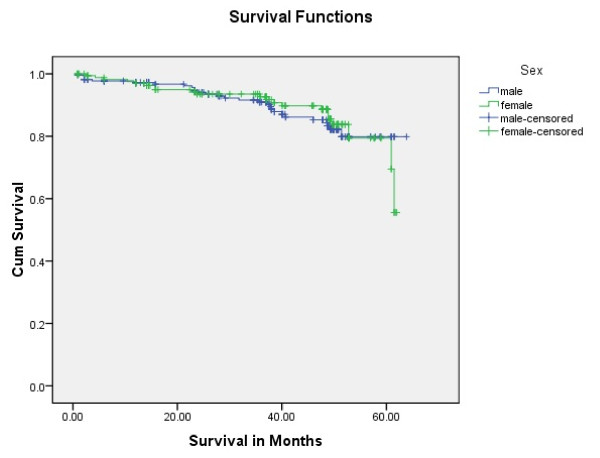
**Survival rate of tongue carcinoma in both gender (log rank p = 0.833)**.

**Figure 4:**
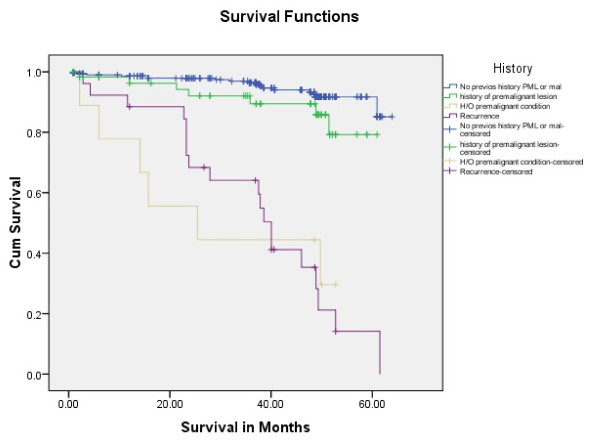
**Survival rate of tongue carcinoma with previous history (log rank p = 0.012)**.

**Figure 5:**
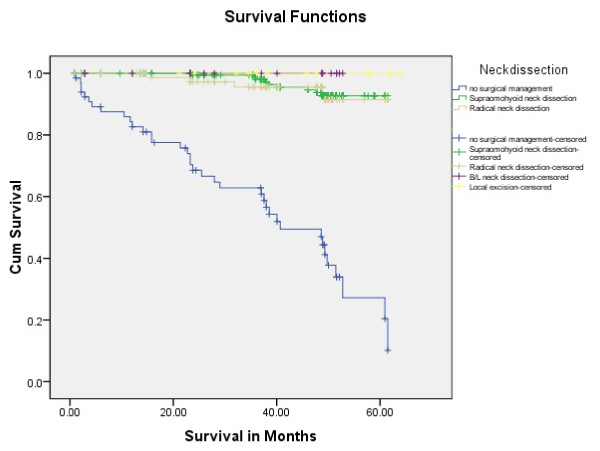
**Survival rate of tongue carcinoma in neck management (log rank p < 0.001)**.

**Figure 6:**
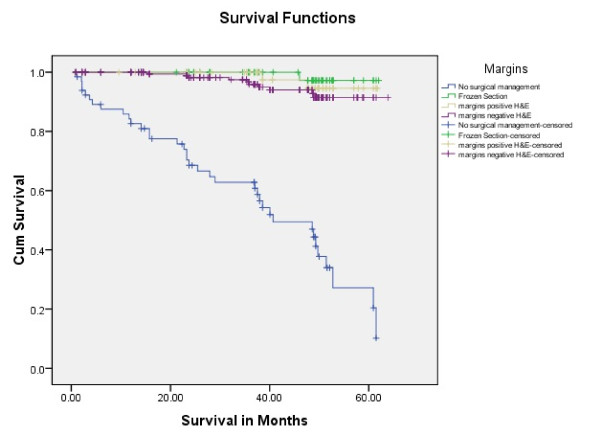
**Survival rate in surgical margin management (log rank p < 0.001)**.

**Figure 7:**
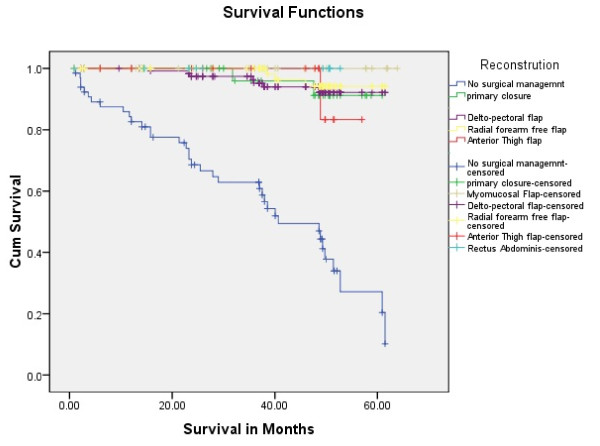
**Survival rate of Tongue carcinoma in different surgical reconstruction**.

**Figure 8:**
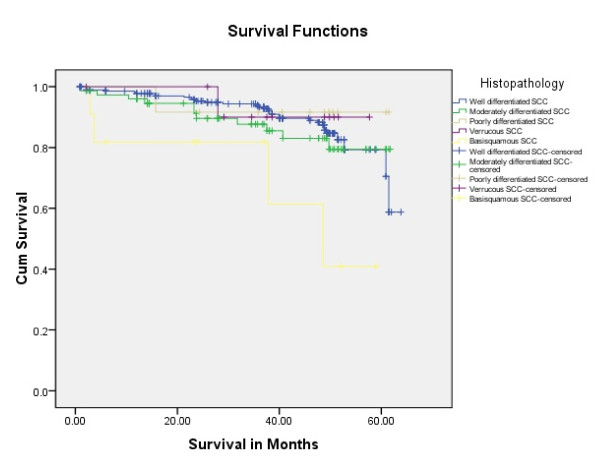
**Survival rate of tongue carcinoma in histopathological variants (log rank p = 0.038)**.

**Figure 9:**
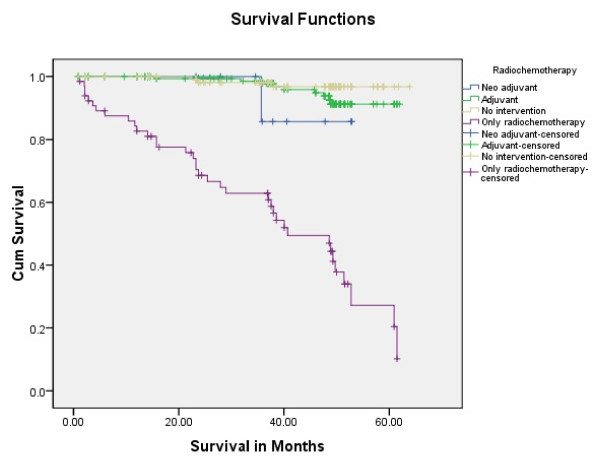
**Survival rate of radio-chemotherapy in tongue carcinoma patients (log rank p < 0.001)**.

Tongue carcinoma patients treated by non surgical treatment modalities had 5 years survival rate of 45.5% and patients with surgical intervention had survival rate of 96.1%.(Log Rank .000) (Figure 1). T1 tumour size had 100% survival rate where as T2, T3, T4 Tumour size had survival rate of 80%,96.7%,77.4% respectively (Log Rank .000).(Figure 2). Survival in both sexes was nearly equal (87.5%, 87.9% Male female)(Log rank .833) (Figure 3). Patients with no previous history of any lesion had maximum survival rate of 94.4% where as recurrent lesion had worst prognosis with survival rate of 26.9% (Figure 4). Patients with bilateral neck dissection had best survival rate of 100% where as supra-omohyoid had survival rate of 95.8% where as patients with no surgical intervention of neck had worst prognosis of 45.5% (Figure 5). Frozen section technique for surgical margin evaluation had survival rate of 98.5% where as patients with no surgical intervention had survival rate of 45.5% (Figure 6). The Myomucosal and Rectus abdominal flap has survival rate of 100% where as Radial forearm free flap has survival rate of 97%.; Delto-pectoral flap has survival rate of 94.9%, primary closure 94.7%, and anterior thigh flap of 93.8% (Figure 7). The well differentiated squamous cell carcinoma had survival rate of 88.9%, moderate differentiated squamous cell carcinoma is 85.5%, poorly differentiated SCC and Verrucous SCC 91.7% each. The Basisquamous SCC survival rate of 63.6% (Figure 8). Survival rate of adjuvant radiochemotherapy was 95.5%, neoadjuvant 90.0% as compared to group of patients treated by radiochemotherapy of 45.5%(Figure 9)

## Discussion

**Figure 10:**
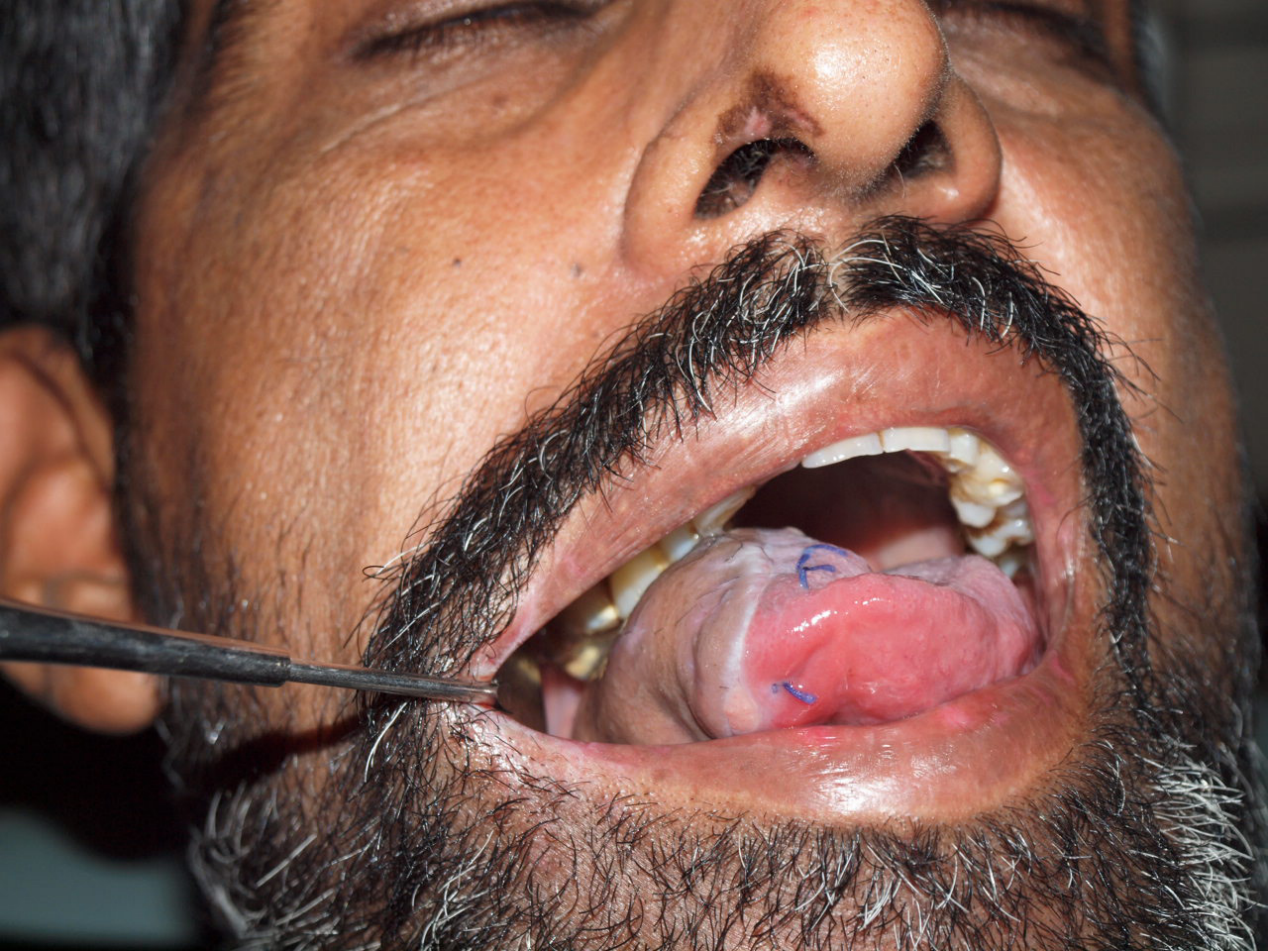
**Reconstructed defect of the toung**. Final result of the radial forearm flap after 2 Weeks.

The various treatment options for Head and Neck Squamous cell carcinoma including tongue carcinoma are surgical, radio-chemotherapy and combination of both. The outcomes of the treatment affect not only the aesthetics but may also compromise the functions of speech swallowing of the suffering patients (Figure 10).

These affects may be of shorter duration or permanent leading to life style changes. The clinician decision for the treatment option depends upon multiple tumour and patient along with health care facilities available.

In this study we evaluated that up to 5 year survival rate was better for the surgical management of tongue carcinoma (96%) as compared to non surgical management (45%) (Table 1). According to literature surgical management has better prognosis [16], [17], [18]. In our study, almost 2.5% of the operated patients received neoadjuvant radiochemotherapy prior to surgery and almost 50% of patients in the surgical group received postoperative radiation due to unclear margins, extensive tumour growth at the primary site, massive lymph node involvement or extracapsular spread, reflecting the scope of changing indications for radiotherapy during a period of three decades. Due to medical almost 17% had no surgical management but only radiochemotherapy. Due to non randomized selection we were unable to determine the impact of radiochemotherapy.

The smaller tumour size T has direct prognostic value. "Smaller the tumour size better the prognosis" this statement is generalized for al HNSCC but most appropriate for the tongue cancer [19]. We have the consistent results (Figure 2). The resection defect is smaller so better the reconstruction and functional rehabilitation.

The prognostic pathogenesis of HNSCC including tongue carcinoma is better known today. The impact HPV, field cancerization and pathogenesis of oral premalignant lesion/conditions with malignant potential in tongue carcinoma patients are also affecting the treatment outcomes [20].In our study we have the same results; the patients with no previous history of premalignant lesion, condition and recurrence had better 5 years survival rate as compared to other groups (Figure 4 Log Rank .012).

The management of neck is an important decision for the clinician. In our study up to 5 years survival is better in patients with neck management (Table 4). We have seen that almost 64% with supraomohyoid neck dissection had 5 year survival rate as it was most frequently performed. The N0 status in tongue carcinoma is also requisite for the selective neck dissection [21].

In our study Radial forearm free flap was most frequently performed (almost 25%) as compared to other free flaps with survival rate of 97%; whereas Deltopectoralis pedicled flap was used to reconstruct tongue in almost 35% of patients of tongue carcinoma with upto 5 years of survival rate of 95% (Table 5).

## Conclusions

Radial forearm free flap was most frequently performed (almost 25%) as compared to other free flaps with survival rate of 97%; whereas Deltopectoralis pedicled flap was used to reconstruct tongue in almost 35% of patients of tongue carcinoma with upto 5 years of survival rate of 95%. We recommend categorical bilateral neck dissection in order to reliably remove occult lymph node metastases. Adjuvant treatment modalities should be applied more frequently in controlled clinical trials and should generally be implemented in cases with unclear margins and lymphatic spread.

## Clinical relevance

This study provides modern treatment strategies for the tongue carcinoma.

## Conflict of interests statement

The authors declare that they have no competing interests.

## Authors' contributions

MR, AI, RW, MRU, AME and NCG conceived of the study and participated in its design and coordination. MR and AI made substantial contributions to data acquisation and conception of manuscript. MR drafted and designed the manuscript. MR and AI performed the statistical analysis. NCG and AME were involved in revising the manuscript. All authors read and approved the final manuscript.

## Consent statement

Written informed consent was obtained from the patient for publication of this case report and accompanying images. A copy of the written consent is available for review by the Editor-in-Chief of this journal.

## Funding

The article processing charges are funded by the Deutsche Forschungsgemeinschaft (DFG), "Open Acess Publizieren".
